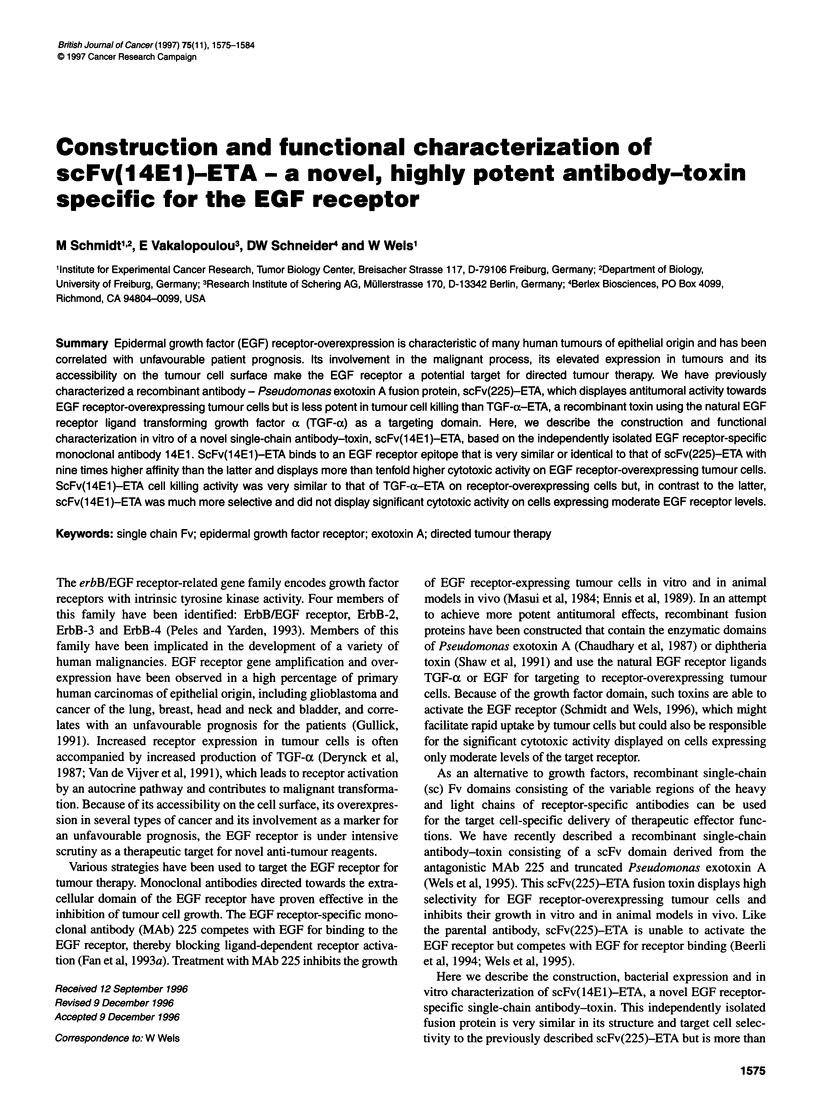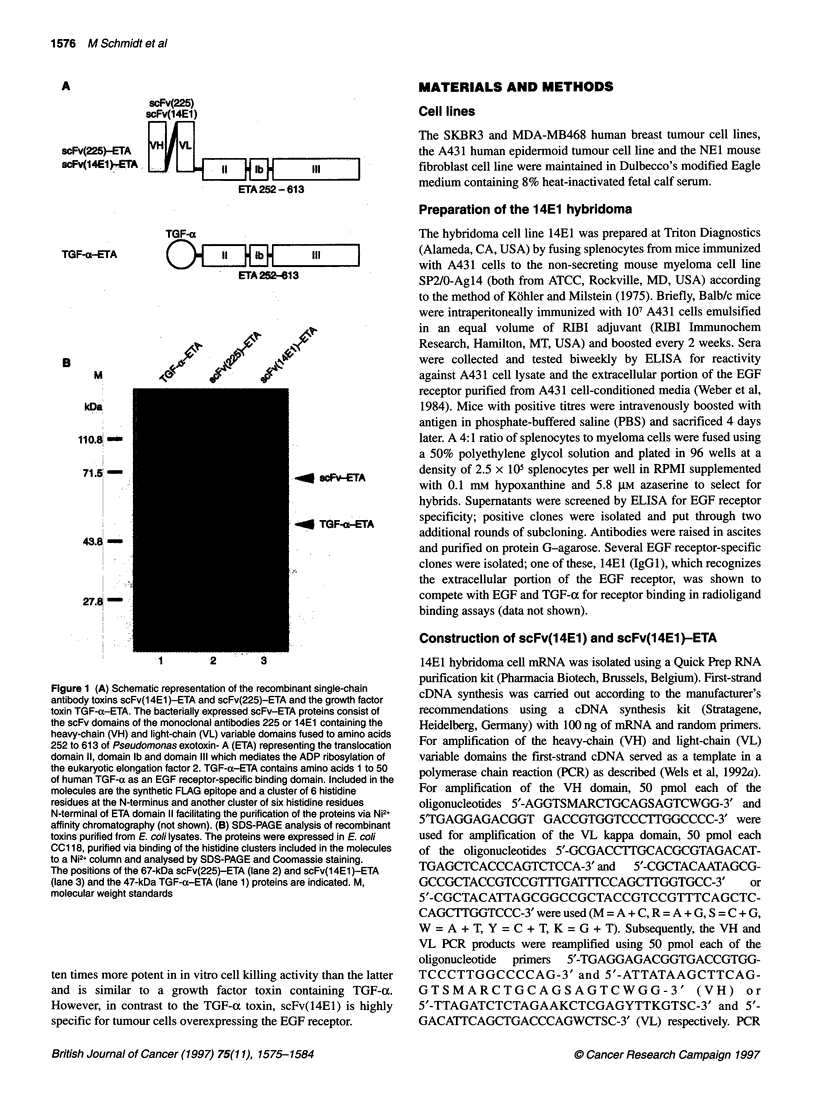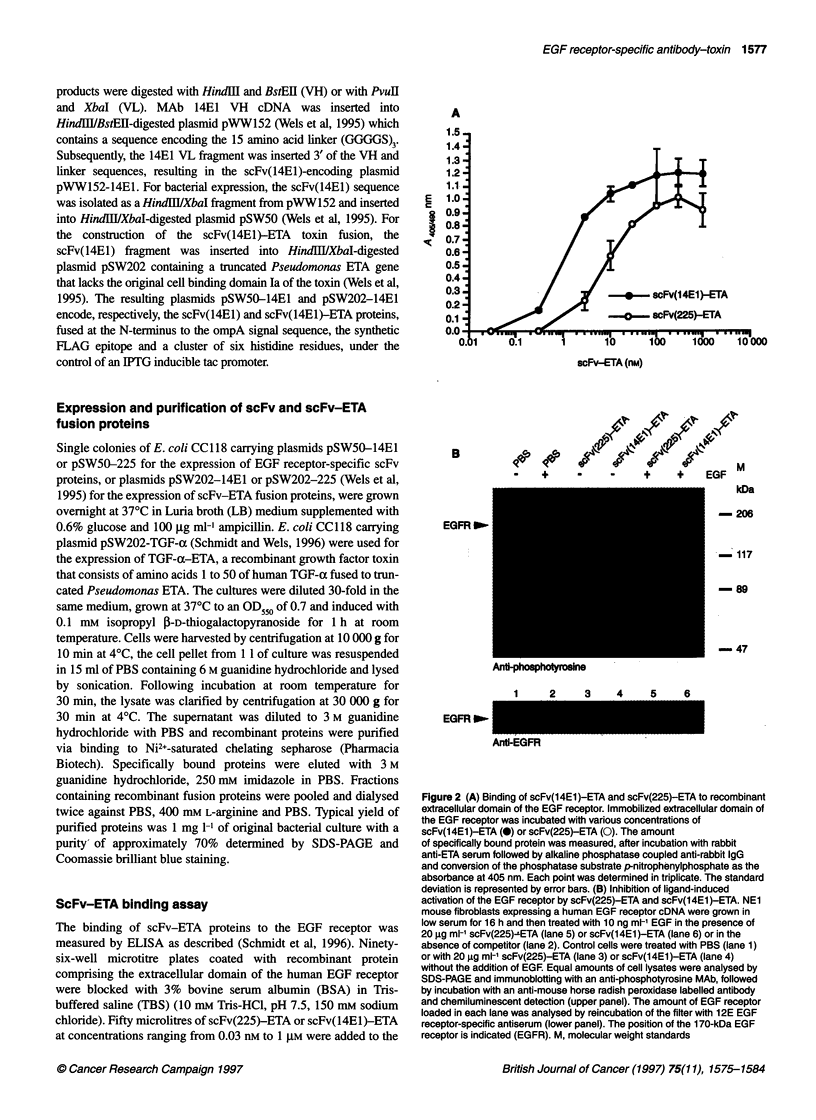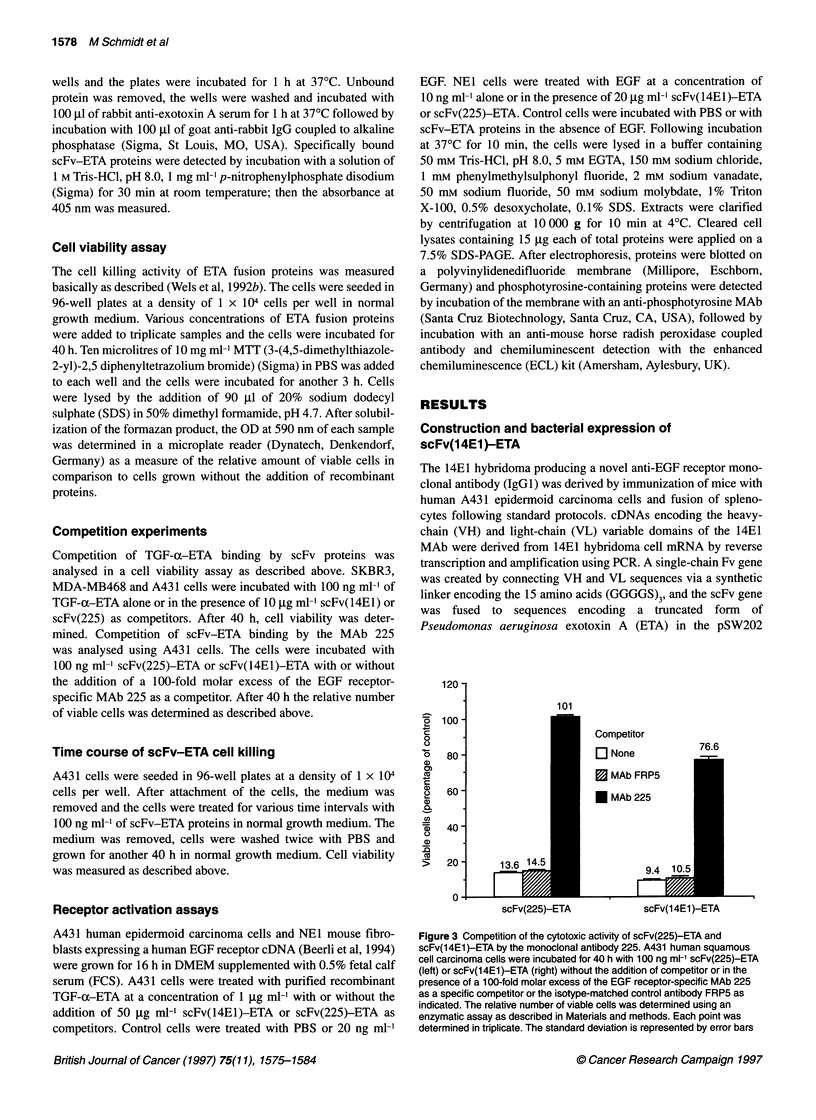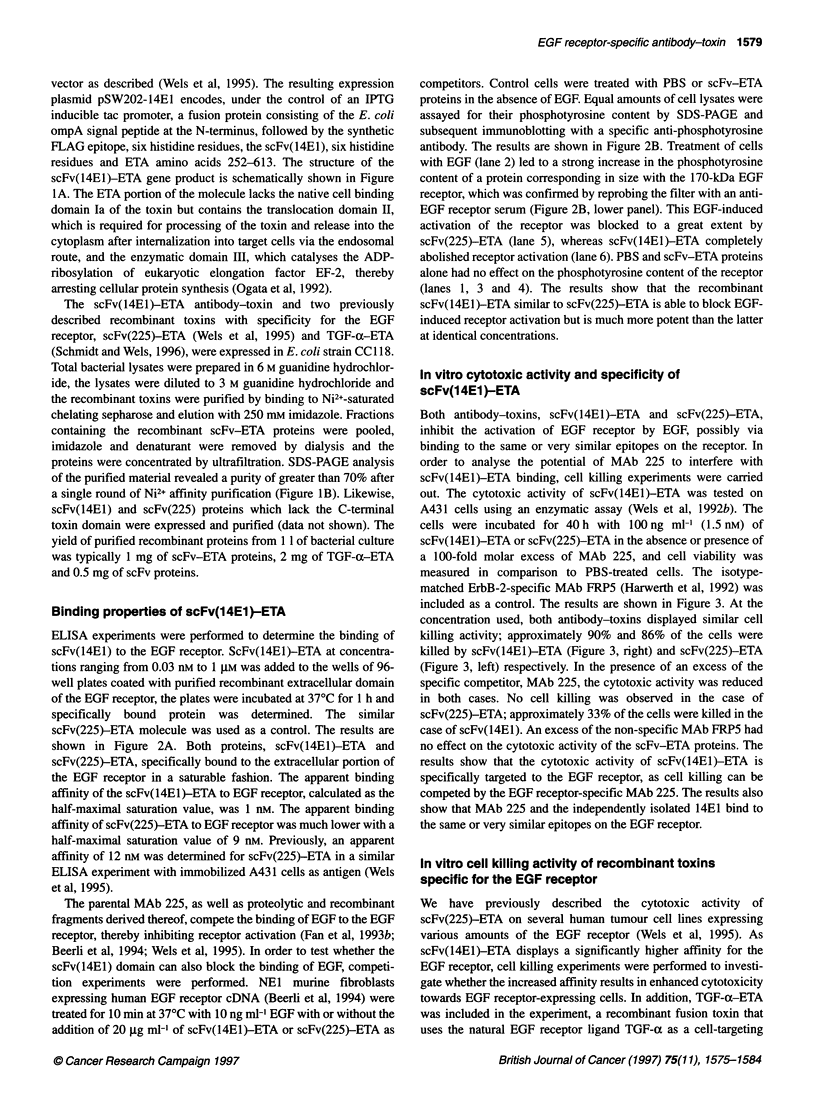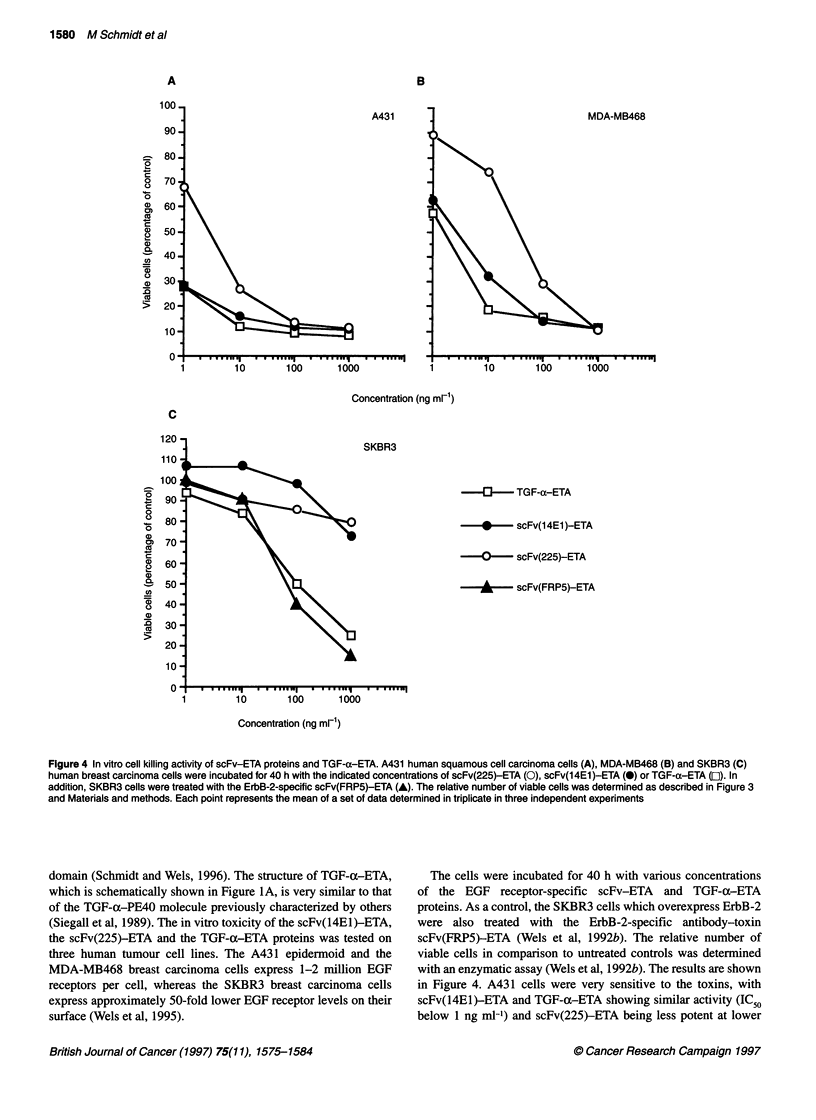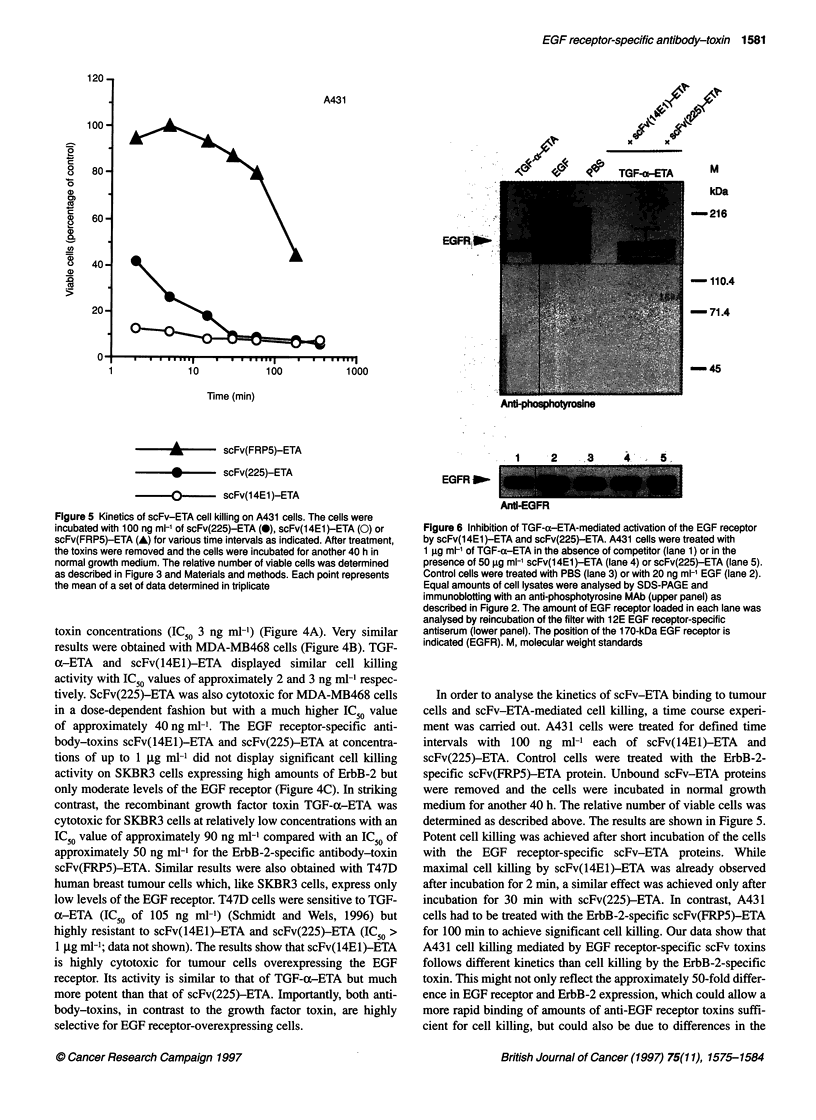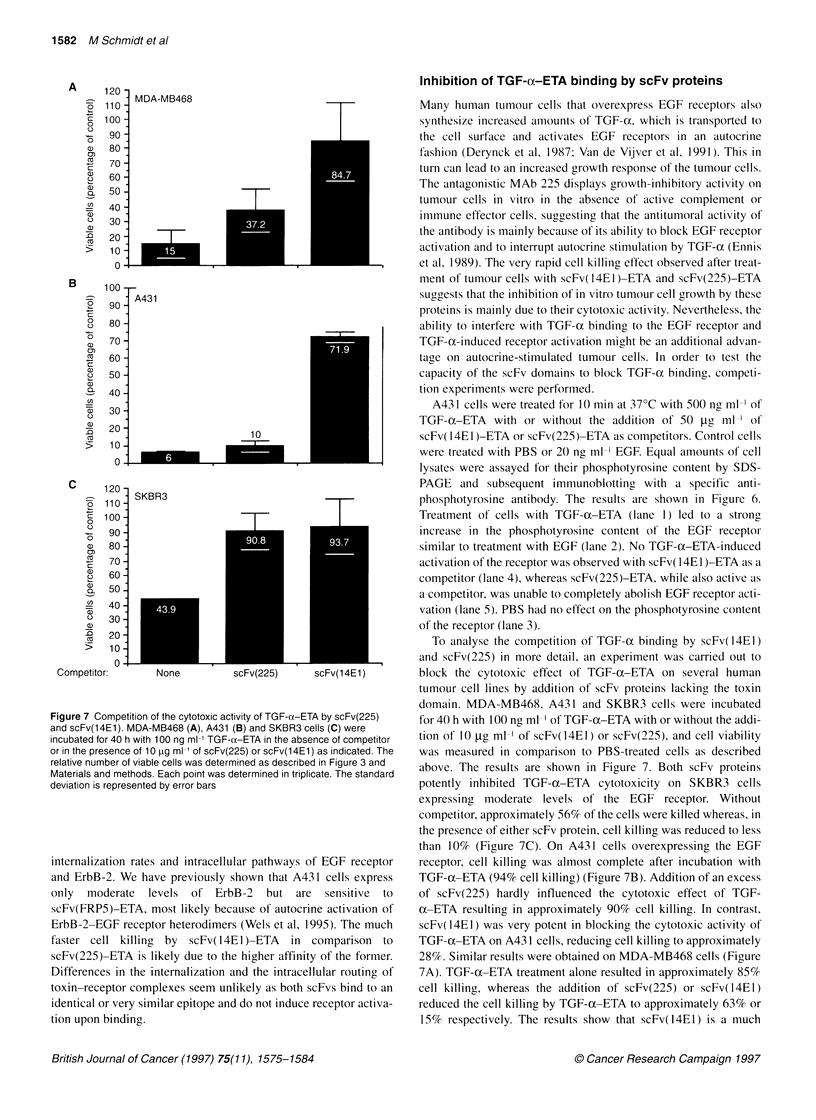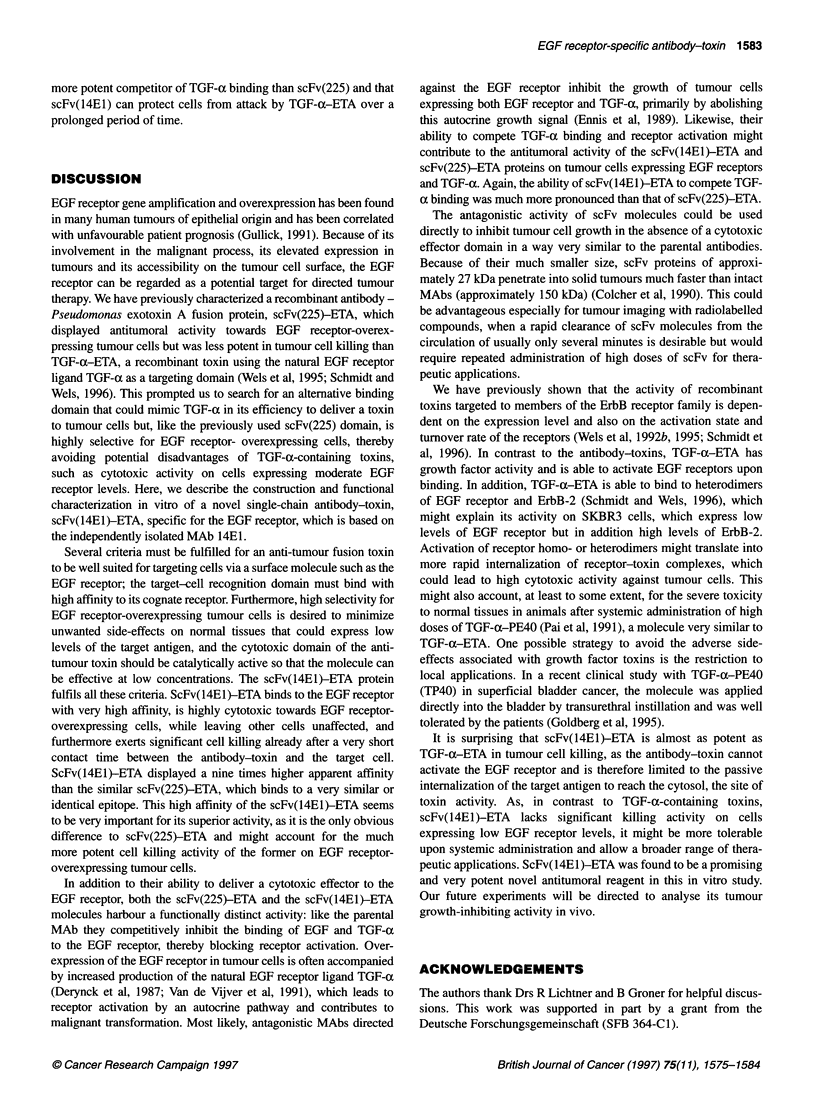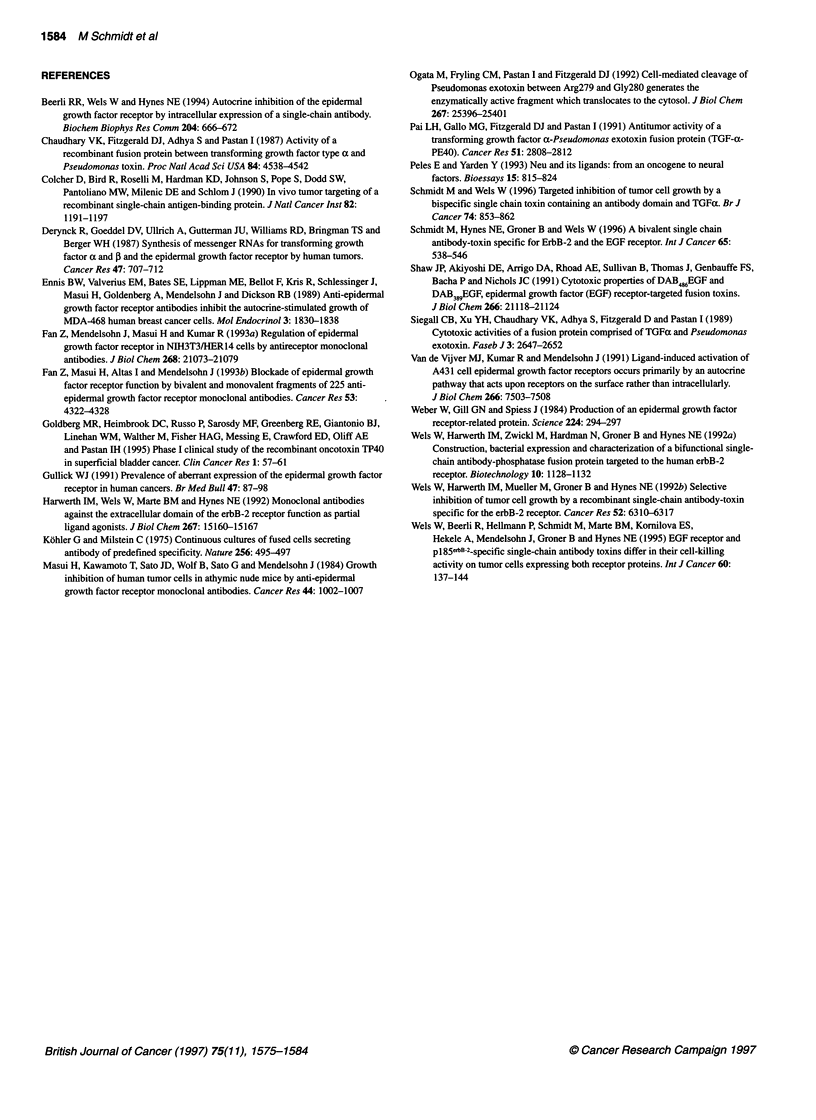# Construction and functional characterization of scFv(14E1)-ETA - a novel, highly potent antibody-toxin specific for the EGF receptor.

**DOI:** 10.1038/bjc.1997.270

**Published:** 1997

**Authors:** M. Schmidt, E. Vakalopoulou, D. W. Schneider, W. Wels

**Affiliations:** Institute for Experimental Cancer Research, Tumor Biology Center, Freiburg, Germany.

## Abstract

**Images:**


*British Journal of Cancer* (1997) **75**(11), 1575–1584
© 1997 Cancer Research Campaign

### Construction and functional characterization of scFv(14E1)-ETA – a novel, highly potent antibody–toxin specific for the EGF receptor

**M Schmidt[1,2], E Vakalopoulou[3], DW Schneider[4] and W Wels[1]**

[1]Institute for Experimental Cancer Research, Tumor Biology Center, Breisacher Strasse 117, D-79106 Freiburg, Germany; [2]Department of Biology, University of Freiburg, Germany; [3]Research Institute of Schering AG, Müllerstrasse 170, D-13342 Berlin, Germany; [4]Berlex Biosciences, PO Box 4099, Richmond, CA 94804–0099, USA

**Summary** Epidermal growth factor (EGF) receptor-overexpression is characteristic of many human tumours of epithelial origin and has been correlated with unfavourable patient prognosis. Its involvement in the malignant process, its elevated expression in tumours and its accessibility on the tumour cell surface make the EGF receptor a potential target for directed tumour therapy. We have previously characterized a recombinant antibody – *Pseudomonas* exotoxin A fusion protein, scFv(225)–ETA, which displayes antitumoral activity towards EGF receptor-overexpressing tumour cells but is less potent in tumour cell killing than TGF-α–ETA, a recombinant toxin using the natural EGF receptor ligand transforming growth factor α (TGF-α) as a targeting domain. Here, we describe the construction and functional characterization in vitro of a novel single-chain antibody–toxin, scFv(14E1)–ETA, based on the independently isolated EGF receptor-specific monoclonal antibody 14E1. ScFv(14E1)–ETA binds to an EGF receptor epitope that is very similar or identical to that of scFv(225)–ETA with nine times higher affinity than the latter and displays more than tenfold higher cytotoxic activity on EGF receptor-overexpressing tumour cells. ScFv(14E1)–ETA cell killing activity was very similar to that of TGF-α–ETA on receptor-overexpressing cells but, in contrast to the latter, scFv(14E1)–ETA was much more selective and did not display significant cytotoxic activity on cells expressing moderate EGF receptor levels.

**Keywords:** single chain Fv; epidermal growth factor receptor; exotoxin A; directed tumour therapy

The *erb*B/EGF receptor-related gene family encodes growth factor receptors with intrinsic tyrosine kinase activity. Four members of this family have been identified: ErbB/EGF receptor, ErbB-2, ErbB-3 and ErbB-4 (Peles and Yarden, 1993). Members of this family have been implicated in the development of a variety of human malignancies. EGF receptor gene amplification and overexpression have been observed in a high percentage of primary human carcinomas of epithelial origin, including glioblastoma and cancer of the lung, breast, head and neck and bladder, and correlates with an unfavourable prognosis for the patients (Gullick, 1991). Increased receptor expression in tumour cells is often accompanied by increased production of TGF-α (Derynck et al, 1987; Van de Vijver et al, 1991), which leads to receptor activation by an autocrine pathway and contributes to malignant transformation. Because of its accessibility on the cell surface, its overexpression in several types of cancer and its involvement as a marker for an unfavourable prognosis, the EGF receptor is under intensive scrutiny as a therapeutic target for novel anti-tumour reagents.

Various strategies have been used to target the EGF receptor for tumour therapy. Monoclonal antibodies directed towards the extracellular domain of the EGF receptor have proven effective in the inhibition of tumour cell growth. The EGF receptor-specific monoclonal antibody (MAb) 225 competes with EGF for binding to the EGF receptor, thereby blocking ligand-dependent receptor activation (Fan et al, 1993*a*). Treatment with MAb 225 inhibits the growth of EGF receptor-expressing tumour cells in vitro and in animal models in vivo (Masui et al, 1984; Ennis et al, 1989). In an attempt to achieve more potent antitumoral effects, recombinant fusion proteins have been constructed that contain the enzymatic domains of *Pseudomonas* exotoxin A (Chaudhary et al, 1987) or diphtheria toxin (Shaw et al, 1991) and use the natural EGF receptor ligands TGF-α or EGF for targeting to receptor-overexpressing tumour cells. Because of the growth factor domain, such toxins are able to activate the EGF receptor (Schmidt and Wels, 1996), which might facilitate rapid uptake by tumour cells but could also be responsible for the significant cytotoxic activity displayed on cells expressing only moderate levels of the target receptor.

As an alternative to growth factors, recombinant single-chain (sc) Fv domains consisting of the variable regions of the heavy and light chains of receptor-specific antibodies can be used for the target cell-specific delivery of therapeutic effector functions. We have recently described a recombinant single-chain antibody–toxin consisting of a scFv domain derived from the antagonistic MAb 225 and truncated *Pseudomonas* exotoxin A (Wels et al, 1995). This scFv(225)–ETA fusion toxin displays high selectivity for EGF receptor-overexpressing tumour cells and inhibits their growth in vitro and in animal models in vivo. Like the parental antibody, scFv(225)–ETA is unable to activate the EGF receptor but competes with EGF for receptor binding (Beerli et al, 1994; Wels et al, 1995).

Here we describe the construction, bacterial expression and in vitro characterization of scFv(14E1)–ETA, a novel EGF receptor-specific single-chain antibody–toxin. This independently isolated fusion protein is very similar in its structure and target cell selectivity to the previously described scFv(225)–ETA but is more than

*Received 12 September 1996*
*Revised 9 December 1996*
*Accepted 9 December 1996*

*Correspondence to:* W Wels

**Figure 1** (**A**) Schematic representation of the recombinant single-chain antibody toxins scFv(14E1)–ETA and scFv(225)–ETA and the growth factor toxin TGF-α–ETA. The bacterially expressed scFv–ETA proteins consist of the scFv domains of the monoclonal antibodies 225 or 14E1 containing the heavy-chain (VH) and light-chain (VL) variable domains fused to amino acids 252 to 613 of *Pseudomonas* exotoxin- A (ETA) representing the translocation domain II, domain Ib and domain III which mediates the ADP ribosylation of the eukaryotic elongation factor 2. TGF-α–ETA contains amino acids 1 to 50 of human TGF-α as an EGF receptor-specific binding domain. Included in the molecules are the synthetic FLAG epitope and a cluster of 6 histidine residues at the N-terminus and another cluster of six histidine residues N-terminal of ETA domain II facilitating the purification of the proteins via $Ni^{2+}$ affinity chromatography (not shown). (**B**) SDS-PAGE analysis of recombinant toxins purified from *E. coli* lysates. The proteins were expressed in *E. coli* CC118, purified via binding of the histidine clusters included in the molecules to a $Ni^{2+}$ column and analysed by SDS-PAGE and Coomassie staining. The positions of the 67-kDa scFv(225)–ETA (lane 2) and scFv(14E1)–ETA (lane 3) and the 47-kDa TGF-α–ETA (lane 1) proteins are indicated. M, molecular weight standards ten times more potent in in vitro cell killing activity than the latter and is similar to a growth factor toxin containing TGF-α. However, in contrast to the TGF-α toxin, scFv(14E1) is highly specific for tumour cells overexpressing the EGF receptor.

### MATERIALS AND METHODS

### Cell lines

The SKBR3 and MDA-MB468 human breast tumour cell lines, the A431 human epidermoid tumour cell line and the NE1 mouse fibroblast cell line were maintained in Dulbecco's modified Eagle medium containing 8% heat-inactivated fetal calf serum.

### Preparation of the 14E1 hybridoma

The hybridoma cell line 14E1 was prepared at Triton Diagnostics (Alameda, CA, USA) by fusing splenocytes from mice immunized with A431 cells to the non-secreting mouse myeloma cell line SP2/0-Ag14 (both from ATCC, Rockville, MD, USA) according to the method of Köhler and Milstein (1975). Briefly, Balb/c mice were intraperitoneally immunized with $10^7$ A431 cells emulsified in an equal volume of RIBI adjuvant (RIBI Immunochem Research, Hamilton, MT, USA) and boosted every 2 weeks. Sera were collected and tested biweekly by ELISA for reactivity against A431 cell lysate and the extracellular portion of the EGF receptor purified from A431 cell-conditioned media (Weber et al, 1984). Mice with positive titres were intravenously boosted with antigen in phosphate-buffered saline (PBS) and sacrificed 4 days later. A 4:1 ratio of splenocytes to myeloma cells were fused using a 50% polyethylene glycol solution and plated in 96 wells at a density of $2.5 \times 10^5$ splenocytes per well in RPMI supplemented with 0.1 mM hypoxanthine and 5.8 μM azaserine to select for hybrids. Supernatants were screened by ELISA for EGF receptor specificity; positive clones were isolated and put through two additional rounds of subcloning. Antibodies were raised in ascites and purified on protein G–agarose. Several EGF receptor-specific clones were isolated; one of these, 14E1 (IgG1), which recognizes the extracellular portion of the EGF receptor, was shown to compete with EGF and TGF-α for receptor binding in radioligand binding assays (data not shown).

### Construction of scFv(14E1) and scFv(14E1)–ETA

14E1 hybridoma cell mRNA was isolated using a Quick Prep RNA purification kit (Pharmacia Biotech, Brussels, Belgium). First-strand cDNA synthesis was carried out according to the manufacturer's recommendations using a cDNA synthesis kit (Stratagene, Heidelberg, Germany) with 100 ng of mRNA and random primers. For amplification of the heavy-chain (VH) and light-chain (VL) variable domains the first-strand cDNA served as a template in a polymerase chain reaction (PCR) as described (Wels et al, 1992*a*). For amplification of the VH domain, 50 pmol each of the oligonucleotides 5′-AGGTSMARCTGCAGSAGTCWGG-3′ and 5′TGAGGAGACGGT GACCGTGGTCCCTTGGCCCC-3′ were used for amplification of the VL kappa domain, 50 pmol each of the oligonucleotides 5′-GCGACCTTGCACGCGTAGACAT-TGAGCTCACCCAGTCTCCA-3′ and 5′-CGCTACAATAGCG-GCCGCTACCGTCCGTTTGATTTCCAGCTTGGTGCC-3′ or 5′-CGCTACATTAGCGGCCGCTACCGTCCGTTTCAGCTC-CAGCTTGGTCCC-3′ were used (M = A + C, R = A + G, S = C + G, W = A + T, Y = C + T, K = G + T). Subsequently, the VH and VL PCR products were reamplified using 50 pmol each of the oligonucleotide primers 5′-TGAGGAGACGGTGACCGTGG-TCCCTTGGCCCCAG-3′ and 5′-ATTATAAGCTTCAG-GTSMARCTGCAGSAGTCWGG-3′ (VH) or 5′-TTAGATCTCTAGAAKCTCGAGYTTKGTSC-3′ and 5′-GACATTCAGCTGACCCAGWCTSC-3′ (VL) respectively. PCR

 *© Cancer Research Campaign 1997*

products were digested with *Hind*III and *Bst*EII (VH) or with *Pvu*II and *Xba*I (VL). MAb 14E1 VH cDNA was inserted into *Hind*III/*Bst*EII-digested plasmid pWW152 (Wels et al, 1995) which contains a sequence encoding the 15 amino acid linker $(GGGGS)_3$. Subsequently, the 14E1 VL fragment was inserted 3′ of the VH and linker sequences, resulting in the scFv(14E1)-encoding plasmid pWW152-14E1. For bacterial expression, the scFv(14E1) sequence was isolated as a *Hind*III/*Xba*I fragment from pWW152 and inserted into *Hind*III/*Xba*I-digested plasmid pSW50 (Wels et al, 1995). For the construction of the scFv(14E1)–ETA toxin fusion, the scFv(14E1) fragment was inserted into *Hind*III/*Xba*I-digested plasmid pSW202 containing a truncated *Pseudomonas* ETA gene that lacks the original cell binding domain Ia of the toxin (Wels et al, 1995). The resulting plasmids pSW50–14E1 and pSW202–14E1 encode, respectively, the scFv(14E1) and scFv(14E1)–ETA proteins, fused at the N-terminus to the ompA signal sequence, the synthetic FLAG epitope and a cluster of six histidine residues, under the control of an IPTG inducible tac promoter.

### Expression and purification of scFv and scFv–ETA fusion proteins

Single colonies of *E. coli* CC118 carrying plasmids pSW50–14E1 or pSW50–225 for the expression of EGF receptor-specific scFv proteins, or plasmids pSW202–14E1 or pSW202–225 (Wels et al, 1995) for the expression of scFv–ETA fusion proteins, were grown overnight at 37°C in Luria broth (LB) medium supplemented with 0.6% glucose and 100 μg $ml^{-1}$ ampicillin. *E. coli* CC118 carrying plasmid pSW202-TGF-α (Schmidt and Wels, 1996) were used for the expression of TGF-α–ETA, a recombinant growth factor toxin that consists of amino acids 1 to 50 of human TGF-α fused to truncated *Pseudomonas* ETA. The cultures were diluted 30-fold in the same medium, grown at 37°C to an $OD_{550}$ of 0.7 and induced with 0.1 mM isopropyl β-D-thiogalactopyranoside for 1 h at room temperature. Cells were harvested by centrifugation at 10 000 **g** for 10 min at 4°C, the cell pellet from 1 l of culture was resuspended in 15 ml of PBS containing 6 M guanidine hydrochloride and lysed by sonication. Following incubation at room temperature for 30 min, the lysate was clarified by centrifugation at 30 000 **g** for 30 min at 4°C. The supernatant was diluted to 3 M guanidine hydrochloride with PBS and recombinant proteins were purified via binding to $Ni^{2+}$-saturated chelating sepharose (Pharmacia Biotech). Specifically bound proteins were eluted with 3 M guanidine hydrochloride, 250 mM imidazole in PBS. Fractions containing recombinant fusion proteins were pooled and dialysed twice against PBS, 400 mM L-arginine and PBS. Typical yield of purified proteins was 1 mg $l^{-1}$ of original bacterial culture with a purity′ of approximately 70% determined by SDS-PAGE and Coomassie brilliant blue staining.

### ScFv–ETA binding assay

The binding of scFv–ETA proteins to the EGF receptor was measured by ELISA as described (Schmidt et al, 1996). Ninety-six-well microtitre plates coated with recombinant protein comprising the extracellular domain of the human EGF receptor were blocked with 3% bovine serum albumin (BSA) in Tris-buffered saline (TBS) (10 mM Tris-HCl, pH 7.5, 150 mM sodium chloride). Fifty microlitres of scFv(225)–ETA or scFv(14E1)–ETA at concentrations ranging from 0.03 nM to 1 μM were added to the

**Figure 2** (**A**) Binding of scFv(14E1)–ETA and scFv(225)–ETA to recombinant extracellular domain of the EGF receptor. Immobilized extracellular domain of the EGF receptor was incubated with various concentrations of scFv(14E1)–ETA (●) or scFv(225)–ETA (○). The amount of specifically bound protein was measured, after incubation with rabbit anti-ETA serum followed by alkaline phosphatase coupled anti-rabbit IgG and conversion of the phosphatase substrate *p*-nitrophenylphosphate as the absorbance at 405 nm. Each point was determined in triplicate. The standard deviation is represented by error bars. (**B**) Inhibition of ligand-induced activation of the EGF receptor by scFv(225)–ETA and scFv(14E1)–ETA. NE1 mouse fibroblasts expressing a human EGF receptor cDNA were grown in low serum for 16 h and then treated with 10 ng $ml^{-1}$ EGF in the presence of 20 μg $ml^{-1}$ scFv(225)–ETA (lane 5) or scFv(14E1)–ETA (lane 6) or in the absence of competitor (lane 2). Control cells were treated with PBS (lane 1) or with 20 μg $ml^{-1}$ scFv(225)–ETA (lane 3) or scFv(14E1)–ETA (lane 4) without the addition of EGF. Equal amounts of cell lysates were analysed by SDS-PAGE and immunoblotting with an anti-phosphotyrosine MAb, followed by incubation with an anti-mouse horse radish peroxidase labelled antibody and chemiluminescent detection (upper panel). The amount of EGF receptor loaded in each lane was analysed by reincubation of the filter with 12E EGF receptor-specific antiserum (lower panel). The position of the 170-kDa EGF receptor is indicated (EGFR). M, molecular weight standards

© Cancer Research Campaign 1997

wells and the plates were incubated for 1 h at 37°C. Unbound protein was removed, the wells were washed and incubated with 100 µl of rabbit anti-exotoxin A serum for 1 h at 37°C followed by incubation with 100 µl of goat anti-rabbit IgG coupled to alkaline phosphatase (Sigma, St Louis, MO, USA). Specifically bound scFv–ETA proteins were detected by incubation with a solution of 1 M Tris-HCl, pH 8.0, 1 mg ml$^{-1}$ *p*-nitrophenylphosphate disodium (Sigma) for 30 min at room temperature; then the absorbance at 405 nm was measured.

### Cell viability assay

The cell killing activity of ETA fusion proteins was measured basically as described (Wels et al, 1992*b*). The cells were seeded in 96-well plates at a density of $1 \times 10^4$ cells per well in normal growth medium. Various concentrations of ETA fusion proteins were added to triplicate samples and the cells were incubated for 40 h. Ten microlitres of 10 mg ml$^{-1}$ MTT (3-(4,5-dimethylthiazole-2-yl)-2,5 diphenyltetrazolium bromide) (Sigma) in PBS was added to each well and the cells were incubated for another 3 h. Cells were lysed by the addition of 90 µl of 20% sodium dodecyl sulphate (SDS) in 50% dimethyl formamide, pH 4.7. After solubilization of the formazan product, the OD at 590 nm of each sample was determined in a microplate reader (Dynatech, Denkendorf, Germany) as a measure of the relative amount of viable cells in comparison to cells grown without the addition of recombinant proteins.

### Competition experiments

Competition of TGF-α–ETA binding by scFv proteins was analysed in a cell viability assay as described above. SKBR3, MDA-MB468 and A431 cells were incubated with 100 ng ml$^{-1}$ of TGF-α–ETA alone or in the presence of 10 µg ml$^{-1}$ scFv(14E1) or scFv(225) as competitors. After 40 h, cell viability was determined. Competition of scFv–ETA binding by the MAb 225 was analysed using A431 cells. The cells were incubated with 100 ng ml$^{-1}$ scFv(225)–ETA or scFv(14E1)–ETA with or without the addition of a 100-fold molar excess of the EGF receptor-specific MAb 225 as a competitor. After 40 h the relative number of viable cells was determined as described above.

### Time course of scFv–ETA cell killing

A431 cells were seeded in 96-well plates at a density of $1 \times 10^4$ cells per well. After attachment of the cells, the medium was removed and the cells were treated for various time intervals with 100 ng ml$^{-1}$ of scFv–ETA proteins in normal growth medium. The medium was removed, cells were washed twice with PBS and grown for another 40 h in normal growth medium. Cell viability was measured as described above.

### Receptor activation assays

A431 human epidermoid carcinoma cells and NE1 mouse fibroblasts expressing a human EGF receptor cDNA (Beerli et al, 1994) were grown for 16 h in DMEM supplemented with 0.5% fetal calf serum (FCS). A431 cells were treated with purified recombinant TGF-α–ETA at a concentration of 1 µg ml$^{-1}$ with or without the addition of 50 µg ml$^{-1}$ scFv(14E1)–ETA or scFv(225)–ETA as competitors. Control cells were treated with PBS or 20 ng ml$^{-1}$ EGF. NE1 cells were treated with EGF at a concentration of 10 ng ml$^{-1}$ alone or in the presence of 20 µg ml$^{-1}$ scFv(14E1)–ETA or scFv(225)–ETA. Control cells were incubated with PBS or with scFv–ETA proteins in the absence of EGF. Following incubation at 37°C for 10 min, the cells were lysed in a buffer containing 50 mM Tris-HCl, pH 8.0, 5 mM EGTA, 150 mM sodium chloride, 1 mM phenylmethylsulphonyl fluoride, 2 mM sodium vanadate, 50 mM sodium fluoride, 50 mM sodium molybdate, 1% Triton X-100, 0.5% desoxycholate, 0.1% SDS. Extracts were clarified by centrifugation at 10 000 **g** for 10 min at 4°C. Cleared cell lysates containing 15 µg each of total proteins were applied on a 7.5% SDS-PAGE. After electrophoresis, proteins were blotted on a polyvinylidenedifluoride membrane (Millipore, Eschborn, Germany) and phosphotyrosine-containing proteins were detected by incubation of the membrane with an anti-phosphotyrosine MAb (Santa Cruz Biotechnology, Santa Cruz, CA, USA), followed by incubation with an anti-mouse horse radish peroxidase coupled antibody and chemiluminescent detection with the enhanced chemiluminescence (ECL) kit (Amersham, Aylesbury, UK).

### RESULTS

### Construction and bacterial expression of scFv(14E1)–ETA

The 14E1 hybridoma producing a novel anti-EGF receptor monoclonal antibody (IgG1) was derived by immunization of mice with human A431 epidermoid carcinoma cells and fusion of splenocytes following standard protocols. cDNAs encoding the heavy-chain (VH) and light-chain (VL) variable domains of the 14E1 MAb were derived from 14E1 hybridoma cell mRNA by reverse transcription and amplification using PCR. A single-chain Fv gene was created by connecting VH and VL sequences via a synthetic linker encoding the 15 amino acids $(GGGGS)_3$, and the scFv gene was fused to sequences encoding a truncated form of *Pseudomonas aeruginosa* exotoxin A (ETA) in the pSW202

**Figure 3** Competition of the cytotoxic activity of scFv(225)–ETA and scFv(14E1)–ETA by the monoclonal antibody 225. A431 human squamous cell carcinoma cells were incubated for 40 h with 100 ng ml$^{-1}$ scFv(225)–ETA (left) or scFv(14E1)–ETA (right) without the addition of competitor or in the presence of a 100-fold molar excess of the EGF receptor-specific MAb 225 as a specific competitor or the isotype-matched control antibody FRP5 as indicated. The relative number of viable cells was determined using an enzymatic assay as described in Materials and methods. Each point was determined in triplicate. The standard deviation is represented by error bars

 *© Cancer Research Campaign 1997*

vector as described (Wels et al, 1995). The resulting expression plasmid pSW202-14E1 encodes, under the control of an IPTG inducible tac promoter, a fusion protein consisting of the *E. coli* ompA signal peptide at the N-terminus, followed by the synthetic FLAG epitope, six histidine residues, the scFv(14E1), six histidine residues and ETA amino acids 252–613. The structure of the scFv(14E1)–ETA gene product is schematically shown in Figure 1A. The ETA portion of the molecule lacks the native cell binding domain Ia of the toxin but contains the translocation domain II, which is required for processing of the toxin and release into the cytoplasm after internalization into target cells via the endosomal route, and the enzymatic domain III, which catalyses the ADP-ribosylation of eukaryotic elongation factor EF-2, thereby arresting cellular protein synthesis (Ogata et al, 1992).

The scFv(14E1)–ETA antibody–toxin and two previously described recombinant toxins with specificity for the EGF receptor, scFv(225)–ETA (Wels et al, 1995) and TGF-α–ETA (Schmidt and Wels, 1996), were expressed in *E. coli* strain CC118. Total bacterial lysates were prepared in 6 M guanidine hydrochloride, the lysates were diluted to 3 M guanidine hydrochloride and the recombinant toxins were purified by binding to $Ni^{2+}$-saturated chelating sepharose and elution with 250 mM imidazole. Fractions containing the recombinant scFv–ETA proteins were pooled, imidazole and denaturant were removed by dialysis and the proteins were concentrated by ultrafiltration. SDS-PAGE analysis of the purified material revealed a purity of greater than 70% after a single round of $Ni^{2+}$ affinity purification (Figure 1B). Likewise, scFv(14E1) and scFv(225) proteins which lack the C-terminal toxin domain were expressed and purified (data not shown). The yield of purified recombinant proteins from 1 l of bacterial culture was typically 1 mg of scFv–ETA proteins, 2 mg of TGF-α–ETA and 0.5 mg of scFv proteins.

### Binding properties of scFv(14E1)–ETA

ELISA experiments were performed to determine the binding of scFv(14E1) to the EGF receptor. ScFv(14E1)–ETA at concentrations ranging from 0.03 nM to 1 μM was added to the wells of 96-well plates coated with purified recombinant extracellular domain of the EGF receptor, the plates were incubated at 37°C for 1 h and specifically bound protein was determined. The similar scFv(225)–ETA molecule was used as a control. The results are shown in Figure 2A. Both proteins, scFv(14E1)–ETA and scFv(225)–ETA, specifically bound to the extracellular portion of the EGF receptor in a saturable fashion. The apparent binding affinity of the scFv(14E1)–ETA to EGF receptor, calculated as the half-maximal saturation value, was 1 nM. The apparent binding affinity of scFv(225)–ETA to EGF receptor was much lower with a half-maximal saturation value of 9 nM. Previously, an apparent affinity of 12 nM was determined for scFv(225)–ETA in a similar ELISA experiment with immobilized A431 cells as antigen (Wels et al, 1995).

The parental MAb 225, as well as proteolytic and recombinant fragments derived thereof, compete the binding of EGF to the EGF receptor, thereby inhibiting receptor activation (Fan et al, 1993*b*; Beerli et al, 1994; Wels et al, 1995). In order to test whether the scFv(14E1) domain can also block the binding of EGF, competition experiments were performed. NE1 murine fibroblasts expressing human EGF receptor cDNA (Beerli et al, 1994) were treated for 10 min at 37°C with 10 ng $ml^{-1}$ EGF with or without the addition of 20 μg $ml^{-1}$ of scFv(14E1)–ETA or scFv(225)–ETA as competitors. Control cells were treated with PBS or scFv–ETA proteins in the absence of EGF. Equal amounts of cell lysates were assayed for their phosphotyrosine content by SDS-PAGE and subsequent immunoblotting with a specific anti-phosphotyrosine antibody. The results are shown in Figure 2B. Treatment of cells with EGF (lane 2) led to a strong increase in the phosphotyrosine content of a protein corresponding in size with the 170-kDa EGF receptor, which was confirmed by reprobing the filter with an anti-EGF receptor serum (Figure 2B, lower panel). This EGF-induced activation of the receptor was blocked to a great extent by scFv(225)–ETA (lane 5), whereas scFv(14E1)–ETA completely abolished receptor activation (lane 6). PBS and scFv–ETA proteins alone had no effect on the phosphotyrosine content of the receptor (lanes 1, 3 and 4). The results show that the recombinant scFv(14E1)–ETA similar to scFv(225)–ETA is able to block EGF-induced receptor activation but is much more potent than the latter at identical concentrations.

### In vitro cytotoxic activity and specificity of scFv(14E1)–ETA

Both antibody–toxins, scFv(14E1)–ETA and scFv(225)–ETA, inhibit the activation of EGF receptor by EGF, possibly via binding to the same or very similar epitopes on the receptor. In order to analyse the potential of MAb 225 to interfere with scFv(14E1)–ETA binding, cell killing experiments were carried out. The cytotoxic activity of scFv(14E1)–ETA was tested on A431 cells using an enzymatic assay (Wels et al, 1992*b*). The cells were incubated for 40 h with 100 ng $ml^{-1}$ (1.5 nM) of scFv(14E1)–ETA or scFv(225)–ETA in the absence or presence of a 100-fold molar excess of MAb 225, and cell viability was measured in comparison to PBS-treated cells. The isotype-matched ErbB-2-specific MAb FRP5 (Harwerth et al, 1992) was included as a control. The results are shown in Figure 3. At the concentration used, both antibody–toxins displayed similar cell killing activity; approximately 90% and 86% of the cells were killed by scFv(14E1)–ETA (Figure 3, right) and scFv(225)–ETA (Figure 3, left) respectively. In the presence of an excess of the specific competitor, MAb 225, the cytotoxic activity was reduced in both cases. No cell killing was observed in the case of scFv(225)–ETA; approximately 33% of the cells were killed in the case of scFv(14E1). An excess of the non-specific MAb FRP5 had no effect on the cytotoxic activity of the scFv–ETA proteins. The results show that the cytotoxic activity of scFv(14E1)–ETA is specifically targeted to the EGF receptor, as cell killing can be competed by the EGF receptor-specific MAb 225. The results also show that MAb 225 and the independently isolated 14E1 bind to the same or very similar epitopes on the EGF receptor.

### In vitro cell killing activity of recombinant toxins specific for the EGF receptor

We have previously described the cytotoxic activity of scFv(225)–ETA on several human tumour cell lines expressing various amounts of the EGF receptor (Wels et al, 1995). As scFv(14E1)–ETA displays a significantly higher affinity for the EGF receptor, cell killing experiments were performed to investigate whether the increased affinity results in enhanced cytotoxicity towards EGF receptor-expressing cells. In addition, TGF-α–ETA was included in the experiment, a recombinant fusion toxin that uses the natural EGF receptor ligand TGF-α as a cell-targeting

© Cancer Research Campaign 1997

**Figure 4** In vitro cell killing activity of scFv–ETA proteins and TGF-α–ETA. A431 human squamous cell carcinoma cells (**A**), MDA-MB468 (**B**) and SKBR3 (**C**) human breast carcinoma cells were incubated for 40 h with the indicated concentrations of scFv(225)–ETA (○), scFv(14E1)–ETA (●) or TGF-α–ETA (□). In addition, SKBR3 cells were treated with the ErbB-2-specific scFv(FRP5)–ETA (▲). The relative number of viable cells was determined as described in Figure 3 and Materials and methods. Each point represents the mean of a set of data determined in triplicate in three independent experiments domain (Schmidt and Wels, 1996). The structure of TGF-α–ETA, which is schematically shown in Figure 1A, is very similar to that of the TGF-α–PE40 molecule previously characterized by others (Siegall et al, 1989). The in vitro toxicity of the scFv(14E1)–ETA, the scFv(225)–ETA and the TGF-α–ETA proteins was tested on three human tumour cell lines. The A431 epidermoid and the MDA-MB468 breast carcinoma cells express 1–2 million EGF receptors per cell, whereas the SKBR3 breast carcinoma cells express approximately 50-fold lower EGF receptor levels on their surface (Wels et al, 1995).

The cells were incubated for 40 h with various concentrations of the EGF receptor-specific scFv–ETA and TGF-α–ETA proteins. As a control, the SKBR3 cells which overexpress ErbB-2 were also treated with the ErbB-2-specific antibody–toxin scFv(FRP5)–ETA (Wels et al, 1992*b*). The relative number of viable cells in comparison to untreated controls was determined with an enzymatic assay (Wels et al, 1992*b*). The results are shown in Figure 4. A431 cells were very sensitive to the toxins, with scFv(14E1)–ETA and TGF-α–ETA showing similar activity ($IC_{50}$ below 1 ng ml$^{-1}$) and scFv(225)–ETA being less potent at lower

© Cancer Research Campaign 1997

**Figure 5** Kinetics of scFv–ETA cell killing on A431 cells. The cells were incubated with 100 ng ml⁻¹ of scFv(225)–ETA (●), scFv(14E1)–ETA (○) or scFv(FRP5)–ETA (▲) for various time intervals as indicated. After treatment, the toxins were removed and the cells were incubated for another 40 h in normal growth medium. The relative number of viable cells was determined as described in Figure 3 and Materials and methods. Each point represents the mean of a set of data determined in triplicate

**Figure 6** Inhibition of TGF-α–ETA-mediated activation of the EGF receptor by scFv(14E1)–ETA and scFv(225)–ETA. A431 cells were treated with 1 μg ml⁻¹ of TGF-α–ETA in the absence of competitor (lane 1) or in the presence of 50 μg ml⁻¹ scFv(14E1)–ETA (lane 4) or scFv(225)–ETA (lane 5). Control cells were treated with PBS (lane 3) or with 20 ng ml⁻¹ EGF (lane 2). Equal amounts of cell lysates were analysed by SDS-PAGE and immunoblotting with an anti-phosphotyrosine MAb (upper panel) as described in Figure 2. The amount of EGF receptor loaded in each lane was analysed by reincubation of the filter with 12E EGF receptor-specific antiserum (lower panel). The position of the 170-kDa EGF receptor is indicated (EGFR). M, molecular weight standards toxin concentrations ($IC_{50}$ 3 ng ml$^{-1}$) (Figure 4A). Very similar results were obtained with MDA-MB468 cells (Figure 4B). TGF-α–ETA and scFv(14E1)–ETA displayed similar cell killing activity with $IC_{50}$ values of approximately 2 and 3 ng ml$^{-1}$ respectively. ScFv(225)–ETA was also cytotoxic for MDA-MB468 cells in a dose-dependent fashion but with a much higher $IC_{50}$ value of approximately 40 ng ml$^{-1}$. The EGF receptor-specific antibody–toxins scFv(14E1)–ETA and scFv(225)–ETA at concentrations of up to 1 μg ml$^{-1}$ did not display significant cell killing activity on SKBR3 cells expressing high amounts of ErbB-2 but only moderate levels of the EGF receptor (Figure 4C). In striking contrast, the recombinant growth factor toxin TGF-α–ETA was cytotoxic for SKBR3 cells at relatively low concentrations with an $IC_{50}$ value of approximately 90 ng ml$^{-1}$ compared with an $IC_{50}$ of approximately 50 ng ml$^{-1}$ for the ErbB-2-specific antibody–toxin scFv(FRP5)–ETA. Similar results were also obtained with T47D human breast tumour cells which, like SKBR3 cells, express only low levels of the EGF receptor. T47D cells were sensitive to TGF-α–ETA ($IC_{50}$ of 105 ng ml$^{-1}$) (Schmidt and Wels, 1996) but highly resistant to scFv(14E1)–ETA and scFv(225)–ETA ($IC_{50}$ > 1 μg ml$^{-1}$; data not shown). The results show that scFv(14E1)–ETA is highly cytotoxic for tumour cells overexpressing the EGF receptor. Its activity is similar to that of TGF-α–ETA but much more potent than that of scFv(225)–ETA. Importantly, both antibody–toxins, in contrast to the growth factor toxin, are highly selective for EGF receptor-overexpressing cells.

In order to analyse the kinetics of scFv–ETA binding to tumour cells and scFv–ETA-mediated cell killing, a time course experiment was carried out. A431 cells were treated for defined time intervals with 100 ng ml$^{-1}$ each of scFv(14E1)–ETA and scFv(225)–ETA. Control cells were treated with the ErbB-2-specific scFv(FRP5)–ETA protein. Unbound scFv–ETA proteins were removed and the cells were incubated in normal growth medium for another 40 h. The relative number of viable cells was determined as described above. The results are shown in Figure 5. Potent cell killing was achieved after short incubation of the cells with the EGF receptor-specific scFv–ETA proteins. While maximal cell killing by scFv(14E1)–ETA was already observed after incubation for 2 min, a similar effect was achieved only after incubation for 30 min with scFv(225)–ETA. In contrast, A431 cells had to be treated with the ErbB-2-specific scFv(FRP5)–ETA for 100 min to achieve significant cell killing. Our data show that A431 cell killing mediated by EGF receptor-specific scFv toxins follows different kinetics than cell killing by the ErbB-2-specific toxin. This might not only reflect the approximately 50-fold difference in EGF receptor and ErbB-2 expression, which could allow a more rapid binding of amounts of anti-EGF receptor toxins sufficient for cell killing, but could also be due to differences in the

© Cancer Research Campaign 1997

**Figure 7** Competition of the cytotoxic activity of TGF-α–ETA by scFv(225) and scFv(14E1). MDA-MB468 (**A**), A431 (**B**) and SKBR3 cells (**C**) were incubated for 40 h with 100 ng ml$^{-1}$ TGF-α–ETA in the absence of competitor or in the presence of 10 μg ml$^{-1}$ of scFv(225) or scFv(14E1) as indicated. The relative number of viable cells was determined as described in Figure 3 and Materials and methods. Each point was determined in triplicate. The standard deviation is represented by error bars internalization rates and intracellular pathways of EGF receptor and ErbB-2. We have previously shown that A431 cells express only moderate levels of ErbB-2 but are sensitive to scFv(FRP5)–ETA, most likely because of autocrine activation of ErbB-2–EGF receptor heterodimers (Wels et al, 1995). The much faster cell killing by scFv(14E1)–ETA in comparison to scFv(225)–ETA is likely due to the higher affinity of the former. Differences in the internalization and the intracellular routing of toxin–receptor complexes seem unlikely as both scFvs bind to an identical or very similar epitope and do not induce receptor activation upon binding.

### Inhibition of TGF-α–ETA binding by scFv proteins

Many human tumour cells that overexpress EGF receptors also synthesize increased amounts of TGF-α, which is transported to the cell surface and activates EGF receptors in an autocrine fashion (Derynck et al, 1987; Van de Vijver et al, 1991). This in turn can lead to an increased growth response of the tumour cells. The antagonistic MAb 225 displays growth-inhibitory activity on tumour cells in vitro in the absence of active complement or immune effector cells, suggesting that the antitumoral activity of the antibody is mainly because of its ability to block EGF receptor activation and to interrupt autocrine stimulation by TGF-α (Ennis et al, 1989). The very rapid cell killing effect observed after treatment of tumour cells with scFv(14E1)–ETA and scFv(225)–ETA suggests that the inhibition of in vitro tumour cell growth by these proteins is mainly due to their cytotoxic activity. Nevertheless, the ability to interfere with TGF-α binding to the EGF receptor and TGF-α-induced receptor activation might be an additional advantage on autocrine-stimulated tumour cells. In order to test the capacity of the scFv domains to block TGF-α binding, competition experiments were performed.

A431 cells were treated for 10 min at 37°C with 500 ng ml$^{-1}$ of TGF-α–ETA with or without the addition of 50 μg ml$^{-1}$ of scFv(14E1)–ETA or scFv(225)–ETA as competitors. Control cells were treated with PBS or 20 ng ml$^{-1}$ EGF. Equal amounts of cell lysates were assayed for their phosphotyrosine content by SDS-PAGE and subsequent immunoblotting with a specific anti-phosphotyrosine antibody. The results are shown in Figure 6. Treatment of cells with TGF-α–ETA (lane 1) led to a strong increase in the phosphotyrosine content of the EGF receptor similar to treatment with EGF (lane 2). No TGF-α–ETA-induced activation of the receptor was observed with scFv(14E1)–ETA as a competitor (lane 4), whereas scFv(225)–ETA, while also active as a competitor, was unable to completely abolish EGF receptor activation (lane 5). PBS had no effect on the phosphotyrosine content of the receptor (lane 3).

To analyse the competition of TGF-α binding by scFv(14E1) and scFv(225) in more detail, an experiment was carried out to block the cytotoxic effect of TGF-α–ETA on several human tumour cell lines by addition of scFv proteins lacking the toxin domain. MDA-MB468, A431 and SKBR3 cells were incubated for 40 h with 100 ng ml$^{-1}$ of TGF-α–ETA with or without the addition of 10 μg ml$^{-1}$ of scFv(14E1) or scFv(225), and cell viability was measured in comparison to PBS-treated cells as described above. The results are shown in Figure 7. Both scFv proteins potently inhibited TGF-α–ETA cytotoxicity on SKBR3 cells expressing moderate levels of the EGF receptor. Without competitor, approximately 56% of the cells were killed whereas, in the presence of either scFv protein, cell killing was reduced to less than 10% (Figure 7C). On A431 cells overexpressing the EGF receptor, cell killing was almost complete after incubation with TGF-α–ETA (94% cell killing) (Figure 7B). Addition of an excess of scFv(225) hardly influenced the cytotoxic effect of TGF-α–ETA resulting in approximately 90% cell killing. In contrast, scFv(14E1) was very potent in blocking the cytotoxic activity of TGF-α–ETA on A431 cells, reducing cell killing to approximately 28%. Similar results were obtained on MDA-MB468 cells (Figure 7A). TGF-α–ETA treatment alone resulted in approximately 85% cell killing, whereas the addition of scFv(225) or scFv(14E1) reduced the cell killing by TGF-α–ETA to approximately 63% or 15% respectively. The results show that scFv(14E1) is a much

 © Cancer Research Campaign 1997

more potent competitor of TGF-α binding than scFv(225) and that scFv(14E1) can protect cells from attack by TGF-α–ETA over a prolonged period of time.

### DISCUSSION

EGF receptor gene amplification and overexpression has been found in many human tumours of epithelial origin and has been correlated with unfavourable patient prognosis (Gullick, 1991). Because of its involvement in the malignant process, its elevated expression in tumours and its accessibility on the tumour cell surface, the EGF receptor can be regarded as a potential target for directed tumour therapy. We have previously characterized a recombinant antibody – *Pseudomonas* exotoxin A fusion protein, scFv(225)–ETA, which displayed antitumoral activity towards EGF receptor-overexpressing tumour cells but was less potent in tumour cell killing than TGF-α–ETA, a recombinant toxin using the natural EGF receptor ligand TGF-α as a targeting domain (Wels et al, 1995; Schmidt and Wels, 1996). This prompted us to search for an alternative binding domain that could mimic TGF-α in its efficiency to deliver a toxin to tumour cells but, like the previously used scFv(225) domain, is highly selective for EGF receptor- overexpressing cells, thereby avoiding potential disadvantages of TGF-α-containing toxins, such as cytotoxic activity on cells expressing moderate EGF receptor levels. Here, we describe the construction and functional characterization in vitro of a novel single-chain antibody–toxin, scFv(14E1)–ETA, specific for the EGF receptor, which is based on the independently isolated MAb 14E1.

Several criteria must be fulfilled for an anti-tumour fusion toxin to be well suited for targeting cells via a surface molecule such as the EGF receptor; the target–cell recognition domain must bind with high affinity to its cognate receptor. Furthermore, high selectivity for EGF receptor-overexpressing tumour cells is desired to minimize unwanted side-effects on normal tissues that could express low levels of the target antigen, and the cytotoxic domain of the anti-tumour toxin should be catalytically active so that the molecule can be effective at low concentrations. The scFv(14E1)–ETA protein fulfils all these criteria. ScFv(14E1)–ETA binds to the EGF receptor with very high affinity, is highly cytotoxic towards EGF receptor-overexpressing cells, while leaving other cells unaffected, and furthermore exerts significant cell killing already after a very short contact time between the antibody–toxin and the target cell. ScFv(14E1)–ETA displayed a nine times higher apparent affinity than the similar scFv(225)–ETA, which binds to a very similar or identical epitope. This high affinity of the scFv(14E1)–ETA seems to be very important for its superior activity, as it is the only obvious difference to scFv(225)–ETA and might account for the much more potent cell killing activity of the former on EGF receptor-overexpressing tumour cells.

In addition to their ability to deliver a cytotoxic effector to the EGF receptor, both the scFv(225)–ETA and the scFv(14E1)–ETA molecules harbour a functionally distinct activity: like the parental MAb they competitively inhibit the binding of EGF and TGF-α to the EGF receptor, thereby blocking receptor activation. Overexpression of the EGF receptor in tumour cells is often accompanied by increased production of the natural EGF receptor ligand TGF-α (Derynck et al, 1987; Van de Vijver et al, 1991), which leads to receptor activation by an autocrine pathway and contributes to malignant transformation. Most likely, antagonistic MAbs directed against the EGF receptor inhibit the growth of tumour cells expressing both EGF receptor and TGF-α, primarily by abolishing this autocrine growth signal (Ennis et al, 1989). Likewise, their ability to compete TGF-α binding and receptor activation might contribute to the antitumoral activity of the scFv(14E1)–ETA and scFv(225)–ETA proteins on tumour cells expressing EGF receptors and TGF-α. Again, the ability of scFv(14E1)–ETA to compete TGF-α binding was much more pronounced than that of scFv(225)–ETA.

The antagonistic activity of scFv molecules could be used directly to inhibit tumour cell growth in the absence of a cytotoxic effector domain in a way very similar to the parental antibodies. Because of their much smaller size, scFv proteins of approximately 27 kDa penetrate into solid tumours much faster than intact MAbs (approximately 150 kDa) (Colcher et al, 1990). This could be advantageous especially for tumour imaging with radiolabelled compounds, when a rapid clearance of scFv molecules from the circulation of usually only several minutes is desirable but would require repeated administration of high doses of scFv for therapeutic applications.

We have previously shown that the activity of recombinant toxins targeted to members of the ErbB receptor family is dependent on the expression level and also on the activation state and turnover rate of the receptors (Wels et al, 1992*b*, 1995; Schmidt et al, 1996). In contrast to the antibody–toxins, TGF-α–ETA has growth factor activity and is able to activate EGF receptors upon binding. In addition, TGF-α–ETA is able to bind to heterodimers of EGF receptor and ErbB-2 (Schmidt and Wels, 1996), which might explain its activity on SKBR3 cells, which express low levels of EGF receptor but in addition high levels of ErbB-2. Activation of receptor homo- or heterodimers might translate into more rapid internalization of receptor–toxin complexes, which could lead to high cytotoxic activity against tumour cells. This might also account, at least to some extent, for the severe toxicity to normal tissues in animals after systemic administration of high doses of TGF-α–PE40 (Pai et al, 1991), a molecule very similar to TGF-α–ETA. One possible strategy to avoid the adverse side-effects associated with growth factor toxins is the restriction to local applications. In a recent clinical study with TGF-α–PE40 (TP40) in superficial bladder cancer, the molecule was applied directly into the bladder by transurethral instillation and was well tolerated by the patients (Goldberg et al, 1995).

It is surprising that scFv(14E1)–ETA is almost as potent as TGF-α–ETA in tumour cell killing, as the antibody–toxin cannot activate the EGF receptor and is therefore limited to the passive internalization of the target antigen to reach the cytosol, the site of toxin activity. As, in contrast to TGF-α-containing toxins, scFv(14E1)–ETA lacks significant killing activity on cells expressing low EGF receptor levels, it might be more tolerable upon systemic administration and allow a broader range of therapeutic applications. ScFv(14E1)–ETA was found to be a promising and very potent novel antitumoral reagent in this in vitro study. Our future experiments will be directed to analyse its tumour growth-inhibiting activity in vivo.

### ACKNOWLEDGEMENTS

The authors thank Drs R Lichtner and B Groner for helpful discussions. This work was supported in part by a grant from the Deutsche Forschungsgemeinschaft (SFB 364-C1).

© Cancer Research Campaign 1997

### REFERENCES

Beerli RR, Wels W and Hynes NE (1994) Autocrine inhibition of the epidermal growth factor receptor by intracellular expression of a single-chain antibody. *Biochem Biophys Res Comm* **204**: 666–672

Chaudhary VK, Fitzgerald DJ, Adhya S and Pastan I (1987) Activity of a recombinant fusion protein between transforming growth factor type α and *Pseudomonas* toxin. *Proc Natl Acad Sci USA* **84**: 4538–4542

Colcher D, Bird R, Roselli M, Hardman KD, Johnson S, Pope S, Dodd SW, Pantoliano MW, Milenic DE and Schlom J (1990) In vivo tumor targeting of a recombinant single-chain antigen-binding protein. *J Natl Cancer Inst* **82**: 1191–1197

Derynck R, Goeddel DV, Ullrich A, Gutterman JU, Williams RD, Bringman TS and Berger WH (1987) Synthesis of messenger RNAs for transforming growth factor α and β and the epidermal growth factor receptor by human tumors. *Cancer Res* **47**: 707–712

Ennis BW, Valverius EM, Bates SE, Lippman ME, Bellot F, Kris R, Schlessinger J, Masui H, Goldenberg A, Mendelsohn J and Dickson RB (1989) Anti-epidermal growth factor receptor antibodies inhibit the autocrine-stimulated growth of MDA-468 human breast cancer cells. *Mol Endocrinol* **3**: 1830–1838

Fan Z, Mendelsohn J, Masui H and Kumar R (1993*a*) Regulation of epidermal growth factor receptor in NIH3T3/HER14 cells by antireceptor monoclonal antibodies. *J Biol Chem* **268**: 21073–21079

Fan Z, Masui H, Altas I and Mendelsohn J (1993*b*) Blockade of epidermal growth factor receptor function by bivalent and monovalent fragments of 225 anti-epidermal growth factor receptor monoclonal antibodies. *Cancer Res* **53**: 4322–4328

Goldberg MR, Heimbrook DC, Russo P, Sarosdy MF, Greenberg RE, Giantonio BJ, Linehan WM, Walther M, Fisher HAG, Messing E, Crawford ED, Oliff AE and Pastan IH (1995) Phase I clinical study of the recombinant oncotoxin TP40 in superficial bladder cancer. *Clin Cancer Res* **1**: 57–61

Gullick WJ (1991) Prevalence of aberrant expression of the epidermal growth factor receptor in human cancers. *Br Med Bull* **47**: 87–98

Harwerth IM, Wels W, Marte BM and Hynes NE (1992) Monoclonal antibodies against the extracellular domain of the erbB-2 receptor function as partial ligand agonists. *J Biol Chem* **267**: 15160–15167

Köhler G and Milstein C (1975) Continuous cultures of fused cells secreting antibody of predefined specificity. *Nature* **256**: 495–497

Masui H, Kawamoto T, Sato JD, Wolf B, Sato G and Mendelsohn J (1984) Growth inhibition of human tumor cells in athymic nude mice by anti-epidermal growth factor receptor monoclonal antibodies. *Cancer Res* **44**: 1002–1007

Ogata M, Fryling CM, Pastan I and Fitzgerald DJ (1992) Cell-mediated cleavage of Pseudomonas exotoxin between Arg279 and Gly280 generates the enzymatically active fragment which translocates to the cytosol. *J Biol Chem* **267**: 25396–25401

Pai LH, Gallo MG, Fitzgerald DJ and Pastan I (1991) Antitumor activity of a transforming growth factor α-*Pseudomonas* exotoxin fusion protein (TGF-α-PE40). *Cancer Res* **51**: 2808–2812

Peles E and Yarden Y (1993) Neu and its ligands: from an oncogene to neural factors. *Bioessays* **15**: 815–824

Schmidt M and Wels W (1996) Targeted inhibition of tumor cell growth by a bispecific single chain toxin containing an antibody domain and TGFα. *Br J Cancer* **74**: 853–862

Schmidt M, Hynes NE, Groner B and Wels W (1996) A bivalent single chain antibody-toxin specific for ErbB-2 and the EGF receptor. *Int J Cancer* **65**: 538–546

Shaw JP, Akiyoshi DE, Arrigo DA, Rhoad AE, Sullivan B, Thomas J, Genbauffe FS, Bacha P and Nichols JC (1991) Cytotoxic properties of $DAB_{486}$EGF and $DAB_{389}$EGF, epidermal growth factor (EGF) receptor-targeted fusion toxins. *J Biol Chem* **266**: 21118–21124

Siegall CB, Xu YH, Chaudhary VK, Adhya S, Fitzgerald D and Pastan I (1989) Cytotoxic activities of a fusion protein comprised of TGFα and *Pseudomonas* exotoxin. *Faseb J* **3**: 2647–2652

Van de Vijver MJ, Kumar R and Mendelsohn J (1991) Ligand-induced activation of A431 cell epidermal growth factor receptors occurs primarily by an autocrine pathway that acts upon receptors on the surface rather than intracellularly. *J Biol Chem* **266**: 7503–7508

Weber W, Gill GN and Spiess J (1984) Production of an epidermal growth factor receptor-related protein. *Science* **224**: 294–297

Wels W, Harwerth IM, Zwickl M, Hardman N, Groner B and Hynes NE (1992*a*) Construction, bacterial expression and characterization of a bifunctional single-chain antibody-phosphatase fusion protein targeted to the human erbB-2 receptor. *Biotechnology* **10**: 1128–1132

Wels W, Harwerth IM, Mueller M, Groner B and Hynes NE (1992*b*) Selective inhibition of tumor cell growth by a recombinant single-chain antibody-toxin specific for the erbB-2 receptor. *Cancer Res* **52**: 6310–6317

Wels W, Beerli R, Hellmann P, Schmidt M, Marte BM, Kornilova ES, Hekele A, Mendelsohn J, Groner B and Hynes NE (1995) EGF receptor and $p185^{erbB-2}$-specific single-chain antibody toxins differ in their cell-killing activity on tumor cells expressing both receptor proteins. *Int J Cancer* **60**: 137–144

© Cancer Research Campaign 1997